# Associations of Different Phenotypes of Wheezing Illness in Early Childhood with Environmental Variables Implicated in the Aetiology of Asthma

**DOI:** 10.1371/journal.pone.0048359

**Published:** 2012-10-31

**Authors:** Raquel Granell, Jonathan A. C. Sterne, John Henderson

**Affiliations:** School of Social and Community Medicine, University of Bristol, Bristol, United Kingdom; University of Ottawa, Canada

## Abstract

**Rationale:**

Asthma is a complex heterogeneous disease that has increased in prevalence in many industrialised countries. However, the causes of asthma inception remain elusive. Consideration of sub-phenotypes of wheezing may reveal important clues to aetiological risk factors.

**Methods:**

Longitudinal phenotypes capturing population heterogeneity in wheezing reports from birth to 7 years were derived using latent class analysis in the Avon Longitudinal Study of Parents and Children (ALSPAC). Probability of class membership was used to examine the association between five wheezing phenotypes (transient early, prolonged early, intermediate-onset, late-onset, persistent) and early life risk factors for asthma.

**Results:**

Phenotypes had similar patterns and strengths of associations with early environmental factors. Comparing transient early with prolonged early wheezing showed a similar pattern of association with most exposure variables considered in terms of the direction of the effect estimates but with prolonged early wheezing tending to have stronger associations than transient early wheezing except for parity and day care attendance.

**Conclusions:**

Associations with early life risk factors suggested that prolonged early wheeze might be a severe form of transient early wheezing. Although differences were found in the associations of early life risk factors with individual phenotypes, these did not point to novel aetiological pathways. Persistent wheezing phenotype has features suggesting overlap of early and late-onset phenotypes.

## Introduction

Asthma is a complex, heterogeneous disease, which often first manifests in early childhood with wheezing illness. During the 1970 s to 1990 s [1], there was a rise in asthma prevalence in many industrialised countries including the United Kingdom, although it appears that this trend has reached a plateau or even declined more recently [2]. This ‘asthma epidemic’ prompted a vigorous search for potentially modifiable environmental risk factors. Based on a number of epidemiological and experimental observations of pulmonary and immunological development, a major focus of this activity has centred on early life, including the prenatal period [3]. A large number of variables have been considered, including mode of delivery [4], maternal diet [5] early feeding history of the child [5], [6], exposure to allergens [7], [8] and pollutants [9], [10] in early life and the role of infections [11]. Findings are inconsistent and few have more than modest effect sizes. Assuming that the rise in prevalence is not attributable to diagnostic shifts, it would appear to be due either to simultaneous changes in a number of factors with modest effects, or to large effects of one or more factors yet to be identified. Alternatively, some factors may have effects that are specific to subgroups of asthma.

It is now recognised that “asthma” is an overarching term for several disease phenotypes [12], each of which may have different causal pathways in its aetiology. Factors that provoke symptoms of asthma are not necessarily those that are responsible for its inception, and gene-environment interactions may modify the expression of asthma according to different levels of exposure. Therefore, efforts to discover the causes of asthma may be hampered if we focus only on variables that provoke asthma symptoms.

Different phenotypes of wheezing in early childhood (never/infrequent, transient early, prolonged early, intermediate onset, late onset and persistent wheezing), based on longitudinal patterns of reported wheezing, have been identified in a large birth cohort study [13]. All phenotypes except prolonged early wheezing were also identified in an independent cohort with wheezing patterns that were similar in both cohorts [14].

If these phenotypes represent discrete pathophysiological entities, their pattern of associations with early life environmental factors might differ and such differences might reveal clues to their aetiology. Therefore, we examined the associations between wheezing phenotypes in early childhood and environmental variables reported during pregnancy, the perinatal period and during early infancy in a large, British birth cohort recruited during pregnancy, the Avon Longitudinal Study of Parents and Children (ALSPAC).

## Methods

### Ethics Statement

Participants were children born of women recruited to the ALSPAC study during pregnancy. The study methodology has been described elsewhere [15]. Ethical approval for the study was obtained from the ALSPAC Law and Ethics Committee and the Local Research Ethics Committees.

### Wheezing Phenotypes

At 6, 18, 30, 42, 54, 69 and 81 months after birth, mothers were sent a self-completion questionnaire about the health of their child that included questions specific to wheezing. Table S1 in Supporting Information S1 shows the proportion of questionnaires returned and the completeness of data. Presence of wheeze was based on a positive response to either question “In the past 12 months has (your child) had wheezing with whistling on the chest?”, or a report of the occurrence of wheeze within a list of 15 common symptoms. Children’s history of wheezing from 6 months to 7 years was used in a longitudinal latent class analysis to define phenotypes of childhood wheezing as previously described [13]. The best fitting model resulted in six phenotypes: 1. “never/infrequent wheeze ” (68% of children) with approximately 10% prevalence of wheezing at 6 months and declining prevalence of sporadic wheeze thereafter, including children who never reported wheeze; 2. “transient early wheeze” (10%) with 50–60% prevalence of wheeze up to 18 months, declining to low prevalence from 42 months; 3. “prolonged early wheeze” (8%) with peak prevalence of wheeze around 65% at 30 months, declining to low prevalence from 69 months; 4. “intermediate onset wheeze” (2%) with low prevalence of wheeze up to 18 months, rising rapidly to high prevalence from age 42 months; 5. “late onset wheeze” (5%) with approximately 20% prevalence of wheeze up to 42 months, rising to more than 50% prevalence thereafter; and 6. “persistent wheeze” (7%) with 65% prevalence of wheeze at 6 months and approximately 90% prevalence thereafter. Trajectories of prevalence of wheeze for each phenotype are presented in Figure 1 of Henderson et al [13] and Figure 1 of Savenjie & Granell et al [14].

Although children with missing data on wheezing were included in the derivation of wheezing phenotypes and analyses of associations with early life risk factors, phenotype membership is typically less certain for these individuals than for those with complete data. For example, a child for whom wheezing was reported at each observation time would be assigned to the persistent wheezing phenotype with probability close to 1, whereas a child with reports of wheezing at 6 and 18 months, and missing data thereafter, is assigned probabilities 0.83 for transient early wheezing, 0.104 for never/infrequent wheezing, 0.063 for prolonged early wheezing and 0.003 for late onset wheezing. The uncertainty quantified by the posterior probabilities of phenotype membership is accounted for in all analyses of phenotype associations via a weighting procedure. The current manuscript uses results based on analysis of children with at least two responses to questionnaires about wheezing (n = 11,678 children), since these analyses yielded similar results to those based on complete cases (n = 6265 children).

### Early Life Risk Factors

Exposure variables were selected on the basis of either previously reported or theoretical associations with asthma. They were categorised according to timing of exposure: 1) ‘demographic, maternal, pregnancy & child’ (characteristics that do not change over time or were measured before birth), 2) perinatal and 3) postnatal characteristics. The exposure variables were obtained from questionnaires sent to the parents or from health care records. Full details can be found in the online Methods S1 in Supporting Information S1.

### Statistical Methods

In order to minimise the risk of bias due to misclassification, associations of risk factors with wheezing phenotypes were examined using multinomial logistic regression models weighted for the probability of each individual of belonging to each phenotype, These probabilities were estimated previously and referred to as the *posterior probabilities* [13]. For more details, see the online Methods S1 in Supporting Information S1. Crude and adjusted relative risk ratios (also known as multinomial odds ratios) were derived in relation to the never/infrequent wheezing phenotype (reference group). Adjustment was done for all variables in same and previous category as the risk factor of interest. Analysis adjusted for birth weight did not include low birth weight and vice versa. Similarly for duration of gestation and preterm delivery, and neither of these variables was adjusted for birth weight.

Heterogeneity p-values comparing estimate effects across wheezing phenotypes were calculated using Chi-squared tests. Weighted multinomial logistic regression models and tests for heterogeneity were fitted using Stata Version 12.0.

## Results

Table 1 shows the distribution of the exposure variables. A total of 8310 (70.8%) children had complete data on the demographic, maternal, pregnancy and child exposures. Of these children, 4258 (51.2%) were males, 3865 (46.5%) had an asthmatic or allergic mother, 1851 (22.3%) were exposed to maternal smoking during pregnancy and 4593 (55.3%) had at least one sibling. Complete data on perinatal exposures were available on 8107 (69.6%) children. Of these, 308 (3.8%) had low birth weight, 388 (4.8%) were born preterm and 832 (10.3%) were born by caesarean section. Of the 7012 (59.7%) children with complete data on postnatal exposures, 3337 (47.6%) were breastfed for more than 3 months, 1356 (19.3%) were exposed to maternal smoking during the first year and 480 (6.8%) attended day care during the first year.

**Table 1:** Description of study samples in each period with data on wheezing phenotypes.

Demographic, maternal, pregnancy & childcharacteristics (N = 8,310)		Number of children with characteristic (%)
Overcrowding (>0.75 persons per room)		1693 (20.4%)
Gas cooking		4438 (53.4%)
Maternal lower education level±		5009 (60.3%)
Maternal history of asthma or allergy		3865 (46.5%)
Maternal pre-pregnant BMI Mean (SD)		22.9 (3.7)
Maternal smoking during pregnancy		1851 (22.3%)
Maternal use of antibiotics during pregnancy		484 (5.8%)
Gender (male)		4258 (51.2%)
Number of previous pregnancies	*None*	3717 (44.7%)
	*1*	3007 (36.2%)
	*2 or more*	1586 (19.1%)
Rented Housing		1325 (15.9%)
Single mother		1546 (18.6%)
Maternal anxiety at 32 weeks pregnancy	*1^st^ quartile (0–2)*	2412 (29.0%)
	*2^nd^ quartile (3–4)*	1977 (23.8%)
	*3^rd^ quartile (5–7)*	2200 (26.5%)
	*4^th^ quartile (8–16)*	1721 (20.7%)
**Perinatal characteristics (N = 8,107)**		
Maternal age at delivery (years) Mean (SD)		28.9 (4.5)
Birth weight (Kg) Mean (SD)		3.4 (0.5)
Low birth weight (<2.5 Kg)		308 (3.8%)
Pregnancy duration (weeks) Mean (SD)		39.5 (1.7)
Preterm delivery (<37weeks)		388 (4.8%)
Caesarean section		832 (10.3%)
**Postnatal characteristics (N = 7,012)**		
Duration of breast feeding_1	*less than 3 months*	3675 (52.4%)
	*3 months or more*	3337 (47.6%)
Duration of breast feeding_2	*Never*	1455 (20.8%)
	*less than 1 month*	1137 (16.2%)
	*1 to 3 months*	1083 (15.4%)
	*3 to 6 months*	956 (13.6%)
	*6+ months*	2381 (34.0%)
Maternal smoking during first year		1356 (19.3%)
Day care attendance during first year		480 (6.8%)
Family pet ownership during first year		4820 (68.7%)
Maternal anxiety during first year	*1^st^ quartile (0–2)*	2311 (33.0%)
	*2^nd^ quartile (3–4)*	2049 (29.2%)
	*3^rd^ quartile (5–7)*	1173 (16.7%)
	*4^th^ quartile (8–16)*	1479 (21.1%)

± Educated to school leaving certificate at 16 years (GCE level) or lower.

Table 2 shows adjusted associations of demographic, maternal, pregnancy and child characteristics with wheezing phenotypes (crude associations shown in Table S2 in Supporting Information S1). Male gender (adjusted relative risk ratios [aRR] range from 1.31 to 1.74) was associated with a higher risk of each phenotype. Maternal smoking during pregnancy (aRR 1.11 to 1.41) was also associated with a higher risk for all wheezing phenotypes although confidence intervals for the less frequent intermediate and late onset wheezing phenotypes included unity. There was little evidence to support a differential association of these variables between wheezing phenotypes.

**Table 2:** Adjusted associations of demographic, maternal, pregnancy and child characteristics with wheezing phenotypes in ALSPAC.

		Adjusted Relative Risk Ratio (95% CI) for*					
Demographic, maternal, pregnancy & child characteristics (N = 8,310)		Transient early(N = 813)	Prolonged early(N = 553)	Intermediate onset (N = 202)	Late onset(N = 381)	Persistent(N = 587)	HeterogeneityP-value+
Overcrowding		1.06 (0.89, 1.27)	1.06 (0.87, 1.30)	0.78 (0.51, 1.18)	0.93 (0.72, 1.21)	1.12 (0.90, 1.40)	0.51
Gas cooking		1.02 (0.89, 1.17)	1.08 (0.92, 1.26)	0.86 (0.65, 1.15)	1.03 (0.86, 1.23)	1.11 (0.93, 1.32)	0.64
Maternal lower education level±		0.91 (0.79, 1.05)	0.96 (0.82, 1.13)	0.89 (0.66, 1.20)	1.04 (0.86, 1.26)	1.02 (0.84, 1.24)	0.73
Maternal history of asthma or allergy		1.23 (1.08, 1.41)	1.47 (1.26, 1.71)	1.61 (1.21, 2.15)	1.32 (1.10, 1.59)	1.91 (1.60, 2.29)	0.001
Maternal pre-pregnant BMI (per-5 Kg/m^2^)		1.02 (0.93, 1.12)	1.04 (0.94, 1.16)	1.01 (0.83, 1.23)	1.05 (0.93, 1.18)	1.09 (0.97, 1.22)	0.92
Maternal smoking during pregnancy		1.27 (1.07, 1.51)	1.33 (1.10, 1.61)	1.25 (0.87, 1.80)	1.11 (0.88, 1.41)	1.41 (1.14, 1.75)	0.61
Maternal use of antibiotics during pregnancy		1.24 (0.95, 1.63)	1.05 (0.76, 1.45)	1.15 (0.64, 2.07)	1.23 (0.85, 1.77)	1.17 (0.82, 1.65)	0.93
Gender (male)		1.45 (1.27, 1.66)	1.45 (1.24, 1.69)	1.41 (1.06, 1.88)	1.31 (1.10, 1.58)	1.74 (1.46, 2.08)	0.23
Number of previous pregnancies (reference: 0)	*1*	1.24 (1.06, 1.44)	1.16 (0.97, 1.38)	0.89 (0.65, 1.23)	0.85 (0.70, 1.05)	1.56 (1.28, 1.92)	
	*≥2*	1.37 (1.11, 1.68)	1.11 (0.88, 1.41)	0.96 (0.61, 1.51)	0.89 (0.67, 1.19)	1.63 (1.26, 2.12)	2.87×10^−5^
Rented housing		1.06 (0.86, 1.31)	1.27 (1.01, 1.59)	1.30 (0.84, 2.02)	1.21 (0.92, 1.60)	1.69 (1.33, 2.16)	0.04
Single mother		0.94 (0.77, 1.14)	1.05 (0.85, 1.30)	0.84 (0.56, 1.28)	0.91 (0.70, 1.17)	0.90 (0.71, 1.15)	0.77
Maternal anxiety at 32 weeks pregnancy (reference: 1st quartile)	*2^nd^ quartile*	1.20 (0.99, 1.45)	1.16 (0.93, 1.45)	1.26 (0.85, 1.88)	1.24 (0.95, 1.60)	1.48 (1.13, 1.94)	
	*3^rd^ quartile*	1.31 (1.09, 1.57)	1.35 (1.09, 1.67)	1.24 (0.84, 1.84)	1.41 (1.10, 1.80)	1.92 (1.48, 2.48)	
	*4^th^ quartile*	1.61 (1.33, 1.96)	1.84 (1.48, 2.29)	1.46 (0.96, 2.23)	1.53 (1.17, 2.00)	2.55 (1.96, 3.31)	0.01

* compared with never/infrequent wheezing (N = 5774) and using each child’s phenotype probability as weights.

± Educated to school leaving certificate at 16 years (GCE level) or lower.

+ Chi-squared test across phenotypes.

Maternal history of asthma or allergy was associated with a higher risk of each wheezing phenotype (aRR 1.23 to 1.91), and we found evidence that these effects were different among the phenotypes (heterogeneity p-value = 0.001), with intermediate onset (aRR 1.61 95%CI 1.21 to 2.15) and persistent wheezing (aRR 1.91 95%CI 1.60 to 2.29) showing the strongest associations. The risk of transient early (1.37; 1.11–1.68) and persistent (1.63; 1.26–2.12) wheezing was increased among children whose mothers had had previous pregnancies and there was little evidence for association with the other phenotypes (heterogeneity p-value = 2.9×10^−5^). Living in rented housing was also strongly associated with prolonged (1.27; 1.01–1.59) and persistent (1.69; 1.33–2.16) wheezing with little evidence of association with other phenotypes (heterogeneity p-value = 0.04). Prenatal maternal anxiety was associated with all phenotypes (aRR 1.46 to 2.55 for mothers in the highest quartile of anxiety scores). Persistent wheezing had the strongest association (2.55; 1.96–3.31) and intermediate onset had the weakest association (1.46; 0.96–2.23) with high anxiety score in pregnancy; heterogeneity p-value = 0.01.

Table 3 shows adjusted associations between perinatal characteristics and wheezing phenotypes (crude associations shown in Table S3 in Supporting Information S1). Children of low birth weight were more likely to develop intermediate-onset wheezing (2.40; 1.21–4.75), but there was weak evidence of heterogeneity across all wheezing phenotypes (p-value = 0.20). Preterm delivery was associated with prolonged early (1.47; 1.05–2.04) and persistent wheezing (1.49; 1.03–2.16), with weak evidence of heterogeneity across phenotypes (p-value = 0.30).

**Table 3:** Adjusted associations of perinatal characteristics with wheezing phenotypes in ALSPAC.

	Adjusted Relative Risk Ratio (95% CI) for*					
Perinatal characteristicsadjusted by other demographic,maternal, pregnancy, childand perinatal characteristics(N = 8,107)	Transient early(N = 788)	Prolonged early(N = 533)	Intermediateonset (N = 198)	Late onset(N = 375)	Persistent(N = 571)	Heterogeneity P-value+
Maternal age at delivery(per-5-years)	0.92 (0.84, 1.00)	0.87 (0.79, 0.96)	0.91 (0.76, 1.09)	0.98 (0.88, 1.10)	0.90 (0.81, 1.01)	0.58
Birth weight (per-1 Kg)±	1.04 (0.88, 1.22)	0.97 (0.81, 1.16)	0.99 (0.71, 1.39)	0.95 (0.77, 1.18)	0.94 (0.77, 1.16)	0.93
Low birth weight (<2.5 Kg)±	1.00 (0.67, 1.50)	1.05 (0.68, 1.63)	2.40 (1.21, 4.75)	1.31 (0.80, 2.17)	1.04 (0.64, 1.69)	0.20
Pregnancy duration (per-1week)‡	0.98 (0.94, 1.02)	0.97 (0.92, 1.01)	1.04 (0.95, 1.14)	1.02 (0.96, 1.08)	0.93 (0.88, 0.98)	0.08
Preterm delivery (<37 weeks)‡	1.21 (0.89, 1.66)	1.47 (1.05, 2.04)	1.14 (0.59, 2.22)	0.86 (0.54, 1.37)	1.49 (1.03, 2.16)	0.30
Caesarean section	1.02 (0.81, 1.29)	0.99 (0.76, 1.29)	0.94 (0.57, 1.56)	1.19 (0.89, 1.59)	1.03 (0.76, 1.38)	0.87

* compared with never/infrequent wheezing (N = 5642) and using each child’s phenotype probability as weights.

± Not adjusted for each other but adjusted for preterm delivery (<37 weeks).

‡ Not adjusted for each other and not adjusted for birth weight/low birth weight (since birth weight does not influence gestational age).

+ Chi-squared test across phenotypes.

Table 4 shows adjusted associations between postnatal characteristics and wheezing phenotypes (crude associations shown in Table S4 in Supporting Information S1). There was weak evidence for association of breast feeding for at least 3 months with a decreased risk of transient wheezing (0.87; 0.74–1.02), and persistent wheezing (0.79; 0.64–0.98). Conversely, day care attendance in the first year of life was associated with an increased risk of transient early (1.45; 1.10–1.91), prolonged early (1.39; 1.01–1.91) and persistent wheezing (1.22; 0.82–1.82) with little evidence for associations with the other two phenotypes (heterogeneity p-value = 0.07). Postnatal maternal anxiety was similarly associated with all wheezing phenotypes; the strongest associations were with transient (1.51; 1.20–1.92), prolonged early (1.40; 1.07–1.83) and persistent (1.65; 1.21–2.25) wheezing for those mothers in the most anxious group (4th quartile) after adjusting for prenatal maternal anxiety.

**Table 4:** Adjusted associations of postnatal characteristics with wheezing phenotypes in ALSPAC.

			Adjusted Relative Risk Ratio (95% CI) for*					
Postnatal characteristics adjustedby other demographic, maternal, pregnancy,child, perinatal & postnatalcharacteristics (N = 7,012)			Transient early(N = 655)	Prolonged early(N = 469)	Intermediate onset (N = 177)	Late onset(N = 326)	Persistent(N = 470)	HeterogeneityP-value+
Duration of breast feeding_1 (reference:none or <3 months)		*3+ months*	0.87 (0.74, 1.02)	0.93 (0.77, 1.12)	1.00 (0.71, 1.39)	1.02 (0.82, 1.26)	0.79 (0.64, 0.98)	0.46
Duration of breast feeding_2 (reference: none)		*≤1 month*	0.95 (0.74, 1.21)	1.02 (0.77, 1.34)	0.69 (0.40, 1.19)	0.93 (0.67, 1.31)	0.93 (0.68, 1.25)	
		*1 to 3 months*	0.95 (0.74, 1.22)	0.97 (0.73, 1.29)	0.87 (0.52, 1.46)	1.02 (0.73, 1.43)	0.85 (0.62, 1.17)	
		*3 to 6 months*	0.91 (0.70, 1.18)	0.96 (0.71, 1.30)	0.64 (0.35, 1.16)	0.84 (0.58, 1.22)	0.64 (0.44, 0.93)	
		*6+ months*	0.81 (0.64, 1.01)	0.90 (0.70, 1.17)	0.95 (0.60, 1.50)	1.07 (0.80, 1.45)	0.78 (0.58, 1.03)	0.33
Maternal smoking during firstyear			1.12 (0.84, 1.49)	1.20 (0.87, 1.66)	0.86 (0.46, 1.61)	0.87 (0.59, 1.29)	0.93 (0.64, 1.35)	0.58
Day care attendance duringfirst year			1.45 (1.10, 1.91)	1.39 (1.01, 1.91)	0.71 (0.35, 1.44)	0.82 (0.53, 1.26)	1.22 (0.82, 1.82)	0.07
Family pet ownership duringfirst year			1.03 (0.87, 1.21)	1.00 (0.83, 1.20)	0.93 (0.67, 1.31)	0.91 (0.74, 1.13)	1.11 (0.90, 1.38)	0.72
Maternal anxiety during firstyear (reference: 1^st^ quartile)	*2^nd^ quartile*		1.14 (0.94, 1.39)	1.03 (0.82, 1.29)	1.00 (0.67, 1.51)	1.03 (0.79, 1.34)	1.23 (0.94, 1.62)	
	*3^rd^ quartile*		1.19 (0.94, 1.52)	1.15 (0.88, 1.51)	1.09 (0.67, 1.79)	1.36 (1.01, 1.85)	1.35 (0.98, 1.85)	
	*4^th^ quartile*		1.51 (1.20, 1.92)	1.40 (1.07, 1.83)	1.27 (0.78, 2.09)	1.30 (0.95, 1.79)	1.65 (1.21, 2.25)	0.90

* compared with never/infrequent wheezing (N = 4915) and using each child’s phenotype probability as weights.

+ Chi-squared test across phenotypes.

Associations with overcrowding, lower maternal education, having a single mother and parental smoking during the first year of the child’s life were substantially attenuated in the adjusted analyses when compared with the crude analyses. Adjusted associations with maternal smoking during pregnancy, maternal use of antibiotics, rented housing, maternal anxiety during pregnancy and the first year, low birth weight, preterm delivery and duration of breastfeeding were also attenuated when compared with the crude estimates. In contrast, adjusted associations of day care attendance during the first year of the child’s life with wheezing phenotypes were generally stronger than crude associations. There was little evidence for associations of gas cooking, caesarean section and family pet ownership during the first year of the child’s life with wheezing phenotypes.

## Discussion

### Main Results

Our results suggest differences in the associations of risk factors with early- compared with later-onset phenotypes and provide some evidence for heterogeneity of associations between phenotypes for some early life factors, such as maternal parity and day care attendance in the first year. Strength and direction of associations with early life factors were similar for the two transient phenotypes of early onset (TEW and PEW), suggesting similarities in their origins. Association with factors likely to increase the risk of exposure to early respiratory viral infections is compatible with the concept that these phenotypes represent developmental airway abnormalities. Our previously reported finding of greater decrements in maximal mid-expiratory flow at 8 years in prolonged compared with transient early wheeze suggests that this phenotype may be a more severe form of transient early wheezing. We identified few modifiable environmental factors that were strongly associated with phenotypes previously recognised as being linked to asthma and allergy. However, low birth weight was most strongly associated with intermediate onset wheezing, in contrast to the stronger associations of preterm birth with early onset wheezing, whether transient or persistent.

### Interpretation

Martinez showed that reduced lung function soon after birth was associated with recurrent wheezing in infancy [16], [17] and subsequently described the phenotype of transient early wheeze in a cohort of children followed longitudinally from birth [18]. Continued follow up of this cohort also demonstrated tracking of early life lung function to young adulthood [19]. Exposure of infants to tobacco smoke metabolites during pregnancy has been clearly shown to be associated with reduced lung function shortly after birth [20], [21] and subsequently [22], [23], and with increased respiratory symptoms in early life [22]. Abnormal airway development may lead to wheeze in infancy in response to viral infections and other triggers, some of which are associated with markers of social deprivation [24]. Therefore, we expected maternal smoking and socio-economic status to show strong associations with early wheezing phenotypes. We also found a positive association of early wheezing with increased opportunity for exposure to viral respiratory infections through the presence of older siblings (higher parity) and attendance at day care. These factors have been suggested to increase respiratory symptoms in early childhood but to be protective for later asthma and wheezing illness [25]. We found only weak evidence for the latter; both higher parity and daycare were associated with risk ratios for intermediate and late onset wheezing <1 but with confidence intervals including the null.

In considering phenotypes that we had prior reason to believe were associated with asthma and atopy, we have investigated their associations with known asthma risk factors. These associations may therefore simply reflect the distribution of ‘true’ asthma cases among the different phenotype groupings and it may be that a completely different set of influences are involved in the aetiology of component asthma phenotypes. However, there is some justification for this starting point based on what is already known about asthma risk and in attempting to disentangle the importance of risk factors for various components of the asthma phenotype that are differentially expressed in its sub-phenotypes. We found that preterm delivery was strongly associated with prolonged and persistent wheeze, whereas low birth weight showed a different pattern of association towards intermediate and late onset wheeze. Preterm delivery [26] and low birth weight [27] have both been reported to be associated with a higher prevalence of asthma in childhood. Our results suggest that there may be different mechanisms of these associations.

In keeping with our previous report of the lack of association between breastfeeding and asthma in this cohort [6], there was no convincing evidence that breastfeeding was positively or negatively associated with any one wheezing phenotype. The role of early pet exposure in the development of asthma remains unclear. While a protective effect has been reported on subsequent inhalant allergen sensitisation [28], a systematic review suggested a small increased risk of asthma in older children associated with early pet ownership [29]. We found no strong evidence of association of pet ownership overall or specifically cat or dog ownership with any wheezing phenotype in this cohort.

Therefore, early life variables that have been considered as risk or protective factors for asthma in children showed some evidence of association with asthma-related phenotypes in this study but these did not suggest any novel or discrete aetiological pathways associated with any one particular phenotype. Shared risk factors may imply common pathways underpinning disease outcomes, perhaps through influencing an intermediate phenotype. Alternatively, if phenotypes had discrepant associations with risk factors, this might imply differences in the aetiology of the phenotypes concerned, lending weight to the growing evidence that these represent discrete biological entities.

### Strengths & Limitations

One of the strengths of this study is the use of phenotypes that were derived from a population sample using a data-driven approach. This means that preconceptions about phenotype structure, such as presence or absence of atopy, were not influential in selecting phenotype groups. However, this approach gives rise to conceptual difficulties in ascribing characteristics of subjects (risk factors) to disease outcome (phenotype) groups as the latent class approach that we used does not assign individuals to categories but rather assigns a probability of an individual belonging to one or more categories. Therefore, an individual subjects characteristics will be shared around more than one phenotype group, although appropriately weighted by the probability of group membership. This may have attenuated differences between groups but is unlikely to have resulted in spurious associations. A problem common to longitudinal cohort studies is incomplete data acquisition and loss to follow up. In the ALSPAC study, incomplete data is associated with markers of social deprivation, increased exposure to tobacco smoke and increased reported wheeze in infancy in those subsequently lost to follow up. However, we ascertained outcome for a large proportion of the sample and we have no evidence that the structure of associations between exposures and phenotypes is different in those with incomplete data compared with the population included in this analysis. One of the strengths of the ALSPAC study is the ability to consider a large number of variables that have been collected prospectively. Although we have not considered a comprehensive range of early life exposures in seeking associations with phenotypes, we concentrated on those that have been shown to be associated with relevant outcomes in later childhood. As highlighted above, some of the phenotypes are strongly associated with such outcomes as asthma and lung function and could therefore be acting as imprecise proxies for these outcomes. However, this is countered by the differential association of phenotypes with different outcomes so that, for instance persistent wheeze represents a phenotype characterised by reduced lung function, increased bronchial responsiveness and moderate atopy while late onset wheeze has a stronger association with atopy and is less strongly associated with reduced lung function. Therefore, exposures that act primarily through atopic pathways should be expected to be more strongly associated with late onset wheeze and those associated with lung function effects with persistent wheeze.

### Conclusion

In conclusion, our results using phenotypes derived from latent class analysis of wheezing trajectories over time suggest similarities of associations between the two early wheezing phenotypes and the two later-onset wheezing phenotypes, with persistent wheezing variably sharing associations with these two broad categories. Although we have described different strengths of association with asthma-related outcomes for individual wheezing phenotypes, it is likely that other approaches to phenotypic classification will be necessary to further elucidate potential aetiological mechanisms that differ between phenotypes. Such approaches could include markers of airway inflammation, physiological measures, such as early lung function, and genetic variants [30].

## Supporting Information

Supporting Information S1 **Methods S1. Results S1. Table S1** Timing of questionnaires and the proportion of returned questionnaires at each time point. **Table S2** Crude association of demographic, maternal, pregnancy and child characteristics with wheezing phenotypes in ALSPAC. **Table S3** Crude association of perinatal characteristics with wheezing phenotypes in ALSPAC. **Table S4** Crude association of postnatal characteristics with wheezing phenotypes in ALSPAC.
(DOCX)
