# Supplementary material for: Associations of Different Phenotypes of Wheezing Illness in Early Childhood with Environmental Variables Implicated in the Aetiology of Asthma
Source: PLoS One. 2012 Oct 31;7(10):e48359. doi: 10.1371/journal.pone.0048359 (PMC3485223; doi:10.1371/journal.pone.0048359)
Supplement: Supporting Information S1 — Methods S1. Results S1. Table S1 Timing of questionnaires and the proportion of returned questionnaires at each time point. Table S2 Crude association of demographic, maternal, pregnancy and child characteristics with wheezing phenotypes in ALSPAC. Table S3 Crude association of perinatal characteristics with wheezing phenotypes in ALSPAC. Table S4 Crude association of postnatal characteristics with wheezing phenotypes in ALSPAC. (DOCX) [file pone.0048359.s001.docx]

**Online Supporting Information**

Associations of different phenotypes of wheezing illness in early childhood with environmental variables implicated in the aetiology of asthma

Raquel Granell, Jonathan A.C. Sterne & John Henderson

School of Social and Community Medicine, University of Bristol, Bristol, United Kingdom

Correspondence to: Raquel Granell

ALSPAC, Oakfield House

Oakfield Grove, Bristol BS8 2BN

Tel: +44 117 331 0105; Fax: +44 117 331 0080

Email: raquel.granell@bris.ac.uk

**METHODS S1**

# *Early life risk factors*

The exposure variables were categorised according to timing of exposure: 1) ‘demographic, maternal, pregnancy & child’ (characteristics that do not change over time or were measured before birth), 2) perinatal and 3) postnatal characteristics. These were selected on the basis of either previously reported or putative associations with asthma, e.g. through associations with atopy. From questionnaires sent to the mother during pregnancy or 3 to 12 months after delivery, we obtained details of educational attainment (lower level defined as educated to school leaving certificate at 16 years (GCE level in UK) or lower), housing tenure, overcrowding of the home (>0.75 persons per room), gas cooking, marital status, number of previous pregnancies, smoking history and personal history of asthma or allergy. Perinatal data were obtained from delivery health care records and consisted of maternal age at delivery, sex of the child, birth weight (also categorised as low birth weight if <2.5 kilograms) and duration of gestation (also categorised as preterm if <37 weeks completed gestation). Postnatal maternal questionnaires from 3-15 months after birth were used to ascertain breastfeeding history, environmental tobacco smoke exposure, day care attendance and pet ownership. In view of the recent report of a dose-dependent association between maternal anxiety during pregnancy and asthma at age 7½ years [1], the association of prenatal and postnatal self-reported maternal anxiety with early wheezing phenotypes was investigated. Anxiety was measured at 32 weeks gestation and at 8 months after pregnancy using the validated self-report Crown Crisp Experiential Index which ranges from 0 (no anxious) to 16 (very anxious) (4). Maternal anxiety scores were not normally distributed and therefore were converted into 1^st^ quartile [0 to 2], 2^nd^ quartile [3-4], 3^rd^ quartile [5-7] and 4^th^ quartile [8-16]. Each quartile was compared with the lowest quartile (least anxious).

# *Statistical methods*

We used longitudinal latent class analysis, as described in detail previously [2,3], to identify wheezing phenotypes (latent classes) that explain the associations between wheezing at different time points; that is, classes within which the probability of wheezing at each time point is statistically independent of wheeze at other times. A latent class model estimates two sets of parameters: conditional probabilities of wheeze at each time point given membership of a phenotype, and the posterior probabilities of phenotype membership for each child, given their wheezing history.

Associations of risk factors with wheezing phenotypes were examined using multinomial logistic regression models that were weighted according to each individual’s posterior probability of belonging to each phenotype. Children contributed a line of data corresponding to each phenotype with a nonzero posterior probability. For example, a child might have posterior probability 0.9 of persistent wheeze, 0.1 of transient early wheeze and zero for all other phenotypes. In regression analyses, this child would contribute two lines of data, the first for persistent wheeze with weight 0.9 and the second for transient early wheeze with weight 0.1. Relative risk ratios were derived in relation to the never/infrequent wheezing trajectory group, which was used as the reference group in all analyses. We estimated both crude and adjusted relative risk ratios, with adjustment for all variables in same category as the risk factor of interest, and additionally for all variables in preceding categories (ie those that were measured earlier). Analysis adjusted for birth weight did not include low birth weight and vice versa. Analysis adjusted for duration of gestation did not include preterm delivery (and vice versa), and neither of these variables was adjusted for birth weight. Latent class analyses were performed with Mplus 4.1 software (2006, 192 Los Angeles) and weighted multinomial logistic regression models were fitted using Stata Version 11.0.

**RESULTS S1**

Table S1 displays the timing of questionnaires including questions about wheezing and the proportion of returned questionnaires at each time, as well as the proportion of not completed and the proportion of positive and negative responses.

Crude associations between demographic, maternal, pregnancy and child factors and wheezing phenotypes are shown in Table S2. Transient early wheezing (TEW) was associated with maternal history of asthma or allergy, maternal smoking during pregnancy, gender and number of previous pregnancies. TEW was also associated with overcrowding, maternal use of antibiotics and maternal anxiety during pregnancy. Prolonged early wheezing (PEW) was associated with overcrowding, maternal history of asthma or allergy, maternal smoking during pregnancy, gender, rented housing, single mother and anxiety during pregnancy. Intermediate onset wheezing (IOW) was associated with maternal history of asthma or allergy and gender. IOW was also associated with maternal anxiety. Late onset wheezing (LOW) was associated with maternal history of asthma or allergy and gender, also with maternal anxiety during pregnancy. Persistent wheezing (PW) associated with maternal history of asthma or allergy, maternal pre-pregnancy BMI, maternal smoking during pregnancy and gender. PW was also associated with overcrowding, maternal low education level, maternal use of antibiotics, previous pregnancies, rented housing, single mother and maternal anxiety during pregnancy.

Table S3 shows the associations between perinatal characteristics and wheezing phenotypes without any adjusting for confounders. PEW was associated with preterm delivery. IOW was associated with low birth weight. LOW was not associated with any risk factors in this category. PW was associated with preterm delivery.

Table S4 shows the associations between postnatal characteristics and wheezing phenotypes without any adjusting for confounders. TEW was associated with day care during the first year, breastfeeding for at least 3 months, maternal smoking and maternal anxiety during pregnancy. PEW was associated with breastfeeding for at least 3 months, maternal smoking and maternal anxiety during pregnancy. IOW was associated was not associated with any risks factor in this category. LOW was associated with maternal anxiety during pregnancy. PW was associated with breastfeeding for at least 1 month, maternal smoking and anxiety during pregnancy.

Table S1 Timing of questionnaires and the proportion of returned questionnaires at each time point

| Time (months) | Total questionnaires  sent (one per child) | Returned | Not completed | Positive response | Negative response |
| --- | --- | --- | --- | --- | --- |
| 6 | 14,135 | 81% | 0.6% | 26.2% | 73.2% |
| 18 | 14,138 | 78% | 1.1% | 27.2% | 71.7% |
| 30 | 14,138 | 73% | 3.5% | 21.9% | 74.7% |
| 42 | 14,138 | 71% | 0.6% | 17.5% | 81.9% |
| 54 | 12,128 | 79% | 1.3% | 18.7% | 80.0% |
| 69 | 11,268 | 77% | 1.1% | 15.3% | 83.6% |
| 81 | 10,908 | 78% | 1.3% | 13.3% | 85.5% |

Table S2 Crude association of demographic, maternal, pregnancy and child characteristics with wheezing phenotypes in ALSPAC

|  | | Adjusted Relative Risk Ratio (95% CI) for* | | | | |  |
| --- | --- | --- | --- | --- | --- | --- | --- |
| **Demographic, maternal, pregnancy & child characteristics (N=8,310)** | | Transient early  (N=813) | Prolonged early  (N=553) | Intermediate onset (N=202) | Late onset  (N=381) | Persistent  (N=587) | Heterogeneity P-value+ |
| Overcrowding |  | 1.20 (1.02, 1.42) | 1.30 (1.08, 1.55) | 0.83 (0.57, 1.22) | 1.00 (0.79, 1.26) | 1.68 (1.39, 2.04) | 0.001 |
| Gas cooking |  | 1.01 (0.89, 1.16) | 1.04 (0.89, 1.21) | 0.86 (0.64, 1.14) | 1.01 (0.84, 1.21) | 1.04 (0.87, 1.23) | 0.80 |
| Maternal lower education level^±^ | | 0.98 (0.86, 1.12) | 1.06 (0.91, 1.24) | 0.89 (0.67, 1.18) | 1.06 (0.88, 1.27) | 1.22 (1.02, 1.47) | 0.23 |
| Maternal history of asthma or allergy | | 1.25 (1.09, 1.43) | 1.49 (1.28, 1.73) | 1.64 (1.23, 2.18) | 1.34 (1.12, 1.60) | 1.95 (1.63, 2.32) | 0.001 |
| Maternal pre-pregnant BMI (per-5Kg/m2) | | 1.03 (0.94, 1.13) | 1.06 (0.96, 1.17) | 1.01 (0.83, 1.23) | 1.05 (0.93, 1.19) | 1.14 (1.02, 1.28) | 0.64 |
| Maternal smoking during pregnancy | | 1.32 (1.13, 1.54) | 1.51 (1.27, 1.80) | 1.23 (0.88, 1.72) | 1.18 (0.95, 1.46) | 1.77 (1.46, 2.13) | 0.02 |
| Maternal use of antibiotics during pregnancy | | 1.31 (1.00, 1.71) | 1.13 (0.82, 1.57) | 1.18 (0.65, 2.12) | 1.27 (0.88, 1.83) | 1.34 (0.95, 1.88) | 0.94 |
| Gender (male) |  | 1.44 (1.26, 1.65) | 1.43 (1.22, 1.66) | 1.40 (1.05, 1.86) | 1.30 (1.08, 1.55) | 1.71 (1.44, 2.04) | 0.24 |
| Number of previous pregnancies (reference: 0) | *1* | 1.24 (1.07, 1.44) | 1.14 (0.96, 1.35) | 0.89 (0.65, 1.23) | 0.86 (0.70, 1.05) | 1.57 (1.29, 1.92) |  |
|  | *≥2* | 1.42 (1.19, 1.70) | 1.21 (0.98, 1.48) | 0.88 (0.59, 1.31) | 0.90 (0.70, 1.16) | 1.92 (1.53, 2.42) | 1.31×10^-6^ |
| Rented housing |  | 1.18 (0.98, 1.42) | 1.52 (1.25, 1.85) | 1.22 (0.83, 1.79) | 1.23 (0.97, 1.57) | 2.13 (1.74, 2.60) | 4.55×10^-5^ |
| Single mother |  | 1.03 (0.87, 1.23) | 1.30 (1.08, 1.56) | 0.98 (0.68, 1.43) | 1.06 (0.84, 1.34) | 1.26 (1.02, 1.56) | 0.21 |
| Maternal anxiety at 32 weeks pregnancy (reference: 1st quartile) | *2^nd^ quartile* | 1.22 (1.01, 1.48) | 1.20 (0.96, 1.49) | 1.29 (0.87, 1.91) | 1.24 (0.96, 1.61) | 1.57 (1.20, 2.05) |  |
|  | *3^rd^ quartile* | 1.32 (1.10, 1.59) | 1.40 (1.13, 1.73) | 1.28 (0.87, 1.89) | 1.43 (1.12, 1.83) | 2.02 (1.57, 2.61) |  |
|  | *4^th^ quartile* | 1.68 (1.39, 2.04) | 2.04 (1.65, 2.53) | 1.52 (1.01, 2.30) | 1.58 (1.21, 2.06) | 3.06 (2.37, 3.94) | 0.0002 |

* compared with never/infrequent wheezing (N=5774) and using each child’s phenotype probability as weights.

± Educated to school leaving certificate at 16 years (GCE level) or lower

+ Chi-squared test across phenotypes

Table S3 Crude association of perinatal characteristics with wheezing phenotypes in ALSPAC

|  | Adjusted Relative Risk Ratio (95% CI) for* | | | | |  |
| --- | --- | --- | --- | --- | --- | --- |
| **Perinatal characteristics adjusted by other demographic, maternal, pregnancy, child and perinatal characteristics (N=8,107)** | Transient early  (N=788) | Prolonged early  (N=533) | Intermediate onset (N=198) | Late onset  (N=375) | Persistent  (N=571) | Heterogeneity P-value+ |
| Maternal age at delivery (per-5-years) | 0.96 (0.89, 1.03) | 0.87 (0.80, 0.95) | 0.91 (0.78, 1.07) | 0.95 (0.86, 1.05) | 0.89 (0.81, 0.98) | 0.38 |
| Birth weight (per-1Kg) | 1.06 (0.93, 1.21) | 0.96 (0.83, 1.12) | 1.06 (0.80, 1.40) | 0.99 (0.83, 1.18) | 0.93 (0.78, 1.10) | 0.70 |
| Low birth weight (<2.5Kg) | 1.04 (0.72, 1.49) | 1.17 (0.79, 1.74) | 1.85 (1.02, 3.36) | 1.26 (0.80, 1.97) | 1.33 (0.87, 2.02) | 0.54 |
| Pregnancy duration (per-1week) | 0.98 (0.94, 1.02) | 0.96 (0.92, 1.01) | 1.03 (0.94, 1.13) | 1.01 (0.96, 1.07) | 0.92 (0.88, 0.97) | 0.06 |
| Preterm delivery (<37 weeks) | 1.24 (0.91, 1.69) | 1.52 (1.10, 2.10) | 1.18 (0.61, 2.27) | 0.92 (0.58, 1.46) | 1.58 (1.11, 2.27) | 0.29 |
| Caesarean section | 1.00 (0.80, 1.26) | 0.99 (0.76, 1.28) | 0.93 (0.57, 1.51) | 1.20 (0.91, 1.60) | 1.04 (0.78, 1.38) | 0.80 |

* compared with never/infrequent wheezing (N=5642) and using each child’s phenotype probability as weights.

+ Chi-squared test across phenotypes

Table S4 Crude association of postnatal characteristics with wheezing phenotypes in ALSPAC

|  | | | Adjusted Relative Risk Ratio (95% CI) for* | | | | |  |
| --- | --- | --- | --- | --- | --- | --- | --- | --- |
| **Postnatal characteristics adjusted by other demographic, maternal, pregnancy, child, perinatal & postnatal characteristics (N=7,012)** | | | Transient early  (N=655) | Prolonged early  (N=469) | Intermediate onset (N=177) | Late onset  (N=326) | Persistent  (N=470) | Heterogeneity P-value+ |
| Duration of breast feeding_1 (reference: none or <3 months) | | *3+ months* | 0.85 (0.74, 0.99) | 0.85 (0.72, 1.00) | 0.96 (0.71, 1.31) | 0.95 (0.78, 1.16) | 0.68 (0.56, 0.83) | 0.12 |
| Duration of breast feeding_2 (reference: none) | | *≤ 1 month* | 0.92 (0.73, 1.17) | 0.97 (0.74, 1.27) | 0.73 (0.43, 1.24) | 0.96 (0.69, 1.33) | 0.81 (0.61, 1.09) |  |
|  | | *1 to 3 months* | 0.91 (0.71, 1.16) | 0.91 (0.69, 1.20) | 0.90 (0.54, 1.49) | 1.01 (0.73, 1.40) | 0.73 (0.54, 0.99) |  |
|  | | *3 to 6 months* | 0.88 (0.68, 1.12) | 0.87 (0.65, 1.15) | 0.63 (0.35, 1.13) | 0.80 (0.56, 1.14) | 0.51 (0.36, 0.72) |  |
|  | | *6+ months* | 0.78 (0.64, 0.96) | 0.80 (0.63, 1.01) | 0.94 (0.62, 1.42) | 1.00 (0.76, 1.31) | 0.62 (0.48, 0.80) | 0.05 |
| Maternal smoking during first year | | | 1.30 ( 1.09, 1.56) | 1.44 (1.18, 1.75) | 1.03 (0.69, 1.54) | 1.10 (0.86, 1.41) | 1.53 (1.22, 1.91) | 0.17 |
| Day care attendance during first year | | | 1.35 ( 1.04, 1.77) | 1.30 (0.96, 1.77) | 0.77 (0.39, 1.55) | 0.86 (0.56, 1.31) | 1.00 (0.68, 1.46) | 0.17 |
| Family pet ownership during first year | | | 1.02 ( 0.87, 1.19) | 1.03 (0.86, 1.23) | 0.95 (0.68, 1.32) | 0.95 (0.77, 1.17) | 1.13 (0.92, 1.39) | 0.79 |
| Maternal anxiety during first year (reference: 1^st^ quartile) | *2^nd^ quartile* | | 1.20 (0.99, 1.46) | 1.12 (0.90, 1.40) | 1.06 (0.71, 1.57) | 1.08 (0.84, 1.40) | 1.42 (1.09, 1.85) |  |
|  | *3^rd^ quartile* | | 1.33 (1.06, 1.66) | 1.38 (1.07, 1.77) | 1.21 (0.76, 1.92) | 1.52 (1.15, 2.02) | 1.81 (1.35, 2.43) |  |
|  | *4^th^ quartile* | | 1.78 (1.45, 2.18) | 1.86 (1.48, 2.33) | 1.46 (0.96, 2.22) | 1.51 (1.15, 1.99) | 2.54 (1.95, 3.31) | 0.06 |

* compared with never/infrequent wheezing (N=4915) and using each child’s phenotype probability as weights.

+ Chi-squared test across phenotypes

**REFERENCES**

1. Cookson H, Granell R, Joinson C, Ben-Shlomo Y, Henderson AJ (2009) Mothers' anxiety during pregnancy is associated with asthma in their children. JAllergy ClinImmunol 123: 847-853.

2. Henderson J, Granell R, Heron J, Sherriff A, Simpson A, et al. (2008) Associations of wheezing phenotypes in the first six years of life with atopy, lung function and airway responsiveness in mid childhood. Thorax.

3. Savenije OE and Granell R CD, Koppelman GH, Smit HA, Wijga A, et al. (2011) Comparison of childhood wheezing phenotypes in 2 birth cohorts: ALSPAC and PIAMA. J Allergy Clin Immunol 127: 1505-1512.
